# Supplementary material for: Global hypo-methylation in a proportion of glioblastoma enriched for an astrocytic signature is associated with increased invasion and altered immune landscape
Source: eLife. 2022 Nov 22;11:e77335. doi: 10.7554/eLife.77335 (PMC9681209; doi:10.7554/eLife.77335)
Supplement: Figure 2—source data 1. [file elife-77335-fig2-data1.zip › Figure_2_source_data_1/Figure_2B/homerResults/motif24.similar.html]

motif24

## Information for motif24

T
G
C
A
C
T
G
A
T
A
G
C
C
A
T
G
A
T
C
G
C
A
G
T
A
T
C
G
C
G
A
T
C
A
T
G
T
C
A
G
C
A
G
T
C
T
G
A
  
Reverse Opposite:  

G
A
C
T
G
T
C
A
A
G
T
C
G
T
A
C
G
C
T
A
T
A
G
C
G
T
C
A
T
A
G
C
G
A
T
C
A
T
C
G
G
A
C
T
A
C
G
T
  

|  |  |
| --- | --- |
| p-value: | 1e-53 |
| log p-value: | -1.232e+02 |
| Information Content per bp: | 1.460 |
| Number of Target Sequences with motif | 101.0 |
| Percentage of Target Sequences with motif | 4.37% |
| Number of Background Sequences with motif | 2.7 |
| Percentage of Background Sequences with motif | 0.78% |
| Average Position of motif in Targets | 108.2 +/- 52.4bp |
| Average Position of motif in Background | 153.2 +/- 43.5bp |
| Strand Bias (log2 ratio + to - strand density) | -0.5 |
| Multiplicity (# of sites on avg that occur together) | 1.03 |
| Motif File: | file (matrix) reverse opposite |

### Similar de novo motifs found

|  |  |  |  |  |  |  |  |
| --- | --- | --- | --- | --- | --- | --- | --- |
| Rank | Match Score | Redundant Motif | P-value | log P-value | % of Targets | % of Background | Motif file |
| 1 | 0.619 | A C T G A C T G A C G T A C T G A C G T A C G T | 1e-24 | -55.905653 | 7.49% | 3.39% | motif file (matrix) |
